# The antioxidant effects of hydroalcoholic extract of Ashrasi date palm on sperm parameters and DNA fragmentation in diabetic rats

**DOI:** 10.1002/ame2.12222

**Published:** 2022-05-08

**Authors:** Morteza Hosseinipour, Rezvan Asgari, Javid Kermani, Nader Goodarzi, Mitra Bakhtiari

**Affiliations:** ^1^ Faculty of Veterinary Medicine Razi Universtiy Kermanshah Iran; ^2^ Medical Biology Research Center, Health Technology Institute Kermanshah University of Medical Sciences Kermanshah Iran; ^3^ Department of Basic and Pathobiological Sciences, Faculty of Veterinary Medicine Razi Universtiy Kermanshah Iran; ^4^ Fertility and Infertility Research Center, Health Technology Institute Kermanshah University of Medical Sciences Kermanshah Iran

**Keywords:** antioxidant, Ashrasi date palm, diabetes, oxidative stress

## Abstract

**Background:**

Diabetes‐induced oxidative stress can have adverse effects on sperm and its DNA integrity. The Ashrasi date palm (ADP) has potent antioxidant properties. The aim of this study was to evaluate the antioxidant effect of ADP hydroalcoholic extract on sperm parameters and sperm DNA fragmentation in diabetic rats.

**Methods:**

Forty male rats were randomly divided into five groups (*n* = 7): 1, control; 2, diabetic; 3–5, diabetic + ADP (30, 90 and 270 mg/kg for groups 3, 4 and 5, respectively). After preparation of ADP extract and its phytochemical screening, it was administered orally to rats, once a day for 5 weeks. At the end of the study, sperm parameters and sperm DNA fragmentation in all groups were investigated.

**Results:**

At doses of 90 and 270 mg/kg, ADP extract significantly increased the sperm viability compared to diabetic group 2 (*p* = 0.04 and *p* = 0.03, respectively) and resulted in a significant decrease in immotile sperm (*p* = 0.002 and *p* = 0.006, respectively). At a dose of 270 mg/kg, a considerable enhancement of forward sperm motility was observed (*p* = 0.04) and there was a significant decrease in sperm DNA fragmentation (*p* = 0.04).

**Conclusions:**

The findings of the present study show for the first time that the hydroalcoholic extract of ADP has protective and antioxidant effects against diabetes‐induced oxidative stress and can improve sperm parameters and protect sperm DNA integrity.

## Graphical abstract

The findings of present study for the first time revealed that the hydroalcoholic extract of ADP has protective and antioxidant effects against diabetes‐induced oxidative stress. This extract can improve the sperm parameters and protect the sperm DNA integrity.

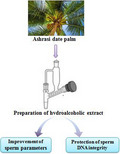

## 1 INTRODUCTION

Diabetes is a metabolic disorder in which the blood glucose level is increased because of a lack of insulin production or a reduction in insulin sensitivity and function.^1^ Diabetes causes injury to different organs and leads to their dysfunctions and failures.^2^ One of the complications of diabetes can be disruption of reproductive system of men.^3^ The metabolism of glucose is a key factor during spermatogenesis while its abnormal homeostasis has detrimental effects on the male reproductive process.^4^ It has been shown that diabetes can have deleterious effects on sperm parameters, chromatin quality and sperm DNA integrity.^4^, ^5^, ^6^, ^7^ Moreover, diabetes induces oxidative stress and can promote the apoptosis process in germ cells.^8^ Under oxidative stress conditions, the levels of reactive oxygen species (ROS) are increased.^9^ High levels of ROS can disturb the normal function of the reproductive system and be a risk factor to the male fertility, and have been shown to lead to cellular injury by overwhelming the capacity of antioxidants.^8^ The enhancement of ROS levels can also lead to peroxidation of the spermatozoa membrane and apoptosis of testicular germ cells, and subsequently affect the sperm parameters.^10^, ^11^ Spermatozoa are sensitive to over‐production of ROS because of loss of a large volume of cytoplasm during spermatogenesis.^12^ Thus, administering various substances with antioxidant properties is an appropriate approach for the reduction of the complications from oxidative stress.^13^, ^14^

A number of studies have investigated the antioxidant effects of different extracts against diabetes‐induced injury in testis and sperm including improvement of sperm parameters, protection of DNA, and reduction of ROS levels.^15^, ^16^, ^17^ Recently, it has been reported that Ashrasi date palm extract has potent antioxidant effects in mercuric chloride‐induced liver destruction and testicular damage.^18^, ^19^ The aqueous date extract has also been shown to play an important role in inhibition of hydroxyl and superoxide radicals.^20^ The antioxidant activity of date extract might be due to phytochemical compounds such as flavonoids, carotenoids and phenolic acids, phytoestrogens, phytosterols and ferulic acid. The date palm also contains steroidal substances or gonadotropin‐like components which can be efficacious in the reproductive system of men.^21^, ^22^ Based on these findings, the Ashrasi date palm may be a source of antioxidant compounds that can improve sperm parameters under diabetes‐induced oxidative stress. Since the literature contains no publications on the antioxidant effects of ADP hydroalcoholic extract on sperm parameters and DNA fragmentation, the present study aimed to evaluate the effect of the ADP extract in diabetic rats.

## 2 METHODS

### 2.1 The preparation of Ashrasi date palm hydroalcoholic extract

Ashrasi date palm fruits were dried for 1 week and then 200 g of dried fruit powder was placed in 2 L of 70% ethanol (1–10 wt/vol), heated for 2 h at 40°C and then cooled. The solution was exposed to ambient temperature for 24 h and then filtered through Whatman No. 2 filter paper. The filtrate was then placed in a rotary vacuum pump for 1 h at 80°C.^18^ Finally, liquid chromatography–mass spectrometry (LC–MS) analysis of the resulting extract of ADP was carried out.

### 2.2 LC–MS analysis of ADP extract

LC–MS analysis was performed according to the protocols and procedures described by Treas and Evans.^23^ Table 1 lists the LC–MS analysis results. Eleven chemical components were identified in the hydroalcoholic extract of ADP, of which the 9‐octadecenoic acid (Z), methyl ester was the most prevalent at 40.95%.^18^

**TABLE 1:** Chemical compounds in hydroalcoholic extract of ADP, determined by LC–MS analysis

Compounds	Retention time (min)	Percentage of the total chromatogram area
Octadecanoic acid, methyl ester	28	5.12
9‐Octadecenoic acid (Z)‐, methyl ester	27.63	40.95
9,12‐Octadecadienoic acid, methyl ester	27.90	7.69
Hexadecanoic acid, methyl ester	23.96	8.95
n‐Butyl laurate	22.11	0.58
Tetradecanoic acid, methyl ester	21.36	7.84
Dodecanoic acid, methyl ester	16.98	20
Phenol, 2,6‐bis (1,1‐di‐methylethyl)‐4‐methyl‐	16.87	2.85
Decanoic acid, methyl ester	13.85	0.82
Octanoic acid, methyl ester	8.33	0.95
Pentanoic acid, 4‐oxo‐, methyl ester	7.14	3.15

Abbreviation: RT, retention time.

### 2.3 Antioxidant activity assessment using 2, 2‐diphenyl‐1‐picrylhydrazyl (DPPH)

The antioxidant activity of ADP hydroalcoholic extract was evaluated using the DPPH free radical method. This assay was performed based on the procedures and protocols explained by Hosseinipour et al.^18^

### 2.4 Animals and design of experiment

Forty male Wistar rats aged 13 weeks old and weighing 240–280 g were purchased from the animal house at Razi University, Iran. The sample size was determined based on similar studies.^10^ The rats were kept under the following conditions: a 12 h/12 h light/dark cycle, relative humidity 50%–55%, temperature 23 ± 1°C. The animals had free access to water and murine food. After 1 week, diabetes was induced in selected rats using a single dose (55 mg/kg bw) of streptozotocin (STZ) introduced by intraperitoneal injection. After 3 days, fasting blood sugar (FBS) in 8 h‐fasted rats was measured with a strip glucometer. Rats with FBS higher than 250 mg/dl were considered as diabetic and included in the study. The FBS of the rats before administration of STZ was 90 mg/dl. The rats were then weighed again and were randomly assigned to 5 groups (8 animals in each group, *n* = 8). The first and second groups—the control and diabetic groups—received 0.5 ml of normal saline/day orally for 5 weeks. The third to fifth groups—the treated diabetic groups—received ADP hydroalcoholic extract at doses of 30, 90 and 270 mg/kg, respectively, orally once a day for 5 weeks.

### 2.5 Hormone assay

At the end of the experimental period, the animals were anesthetized with chloroform and blood samples were collected from the animal’s heart for the evaluation of hormonal concentrations. Then, blood samples were centrifuged at 1000 rpm for 10 min and the isolated serum samples were used to measure the level of testosterone. The level of testosterone was measured using enzyme‐linked immunosorbent assay (ELISA) according to protocol of our previous study.^18^

### 2.6 Sperm preparation

In order to prepare the sperm samples, the rats were killed using deep inhalation of chloroform. The tail of the epididymis was cut and placed in a dish containing 10 ml Ham’s F10 medium where it was crushed using a sterile blade. The dish was incubated in 37°C and 5% CO_2_ for at least 15 min. The released sperm were then used to evaluate the sperm parameters.

### 2.7 Analysis of sperm parameters

The sperm parameters evaluated were sperm count, motility and viability. For the sperm count, 500 μl of the sperm suspension was diluted with 10% formaldehyde (1:10), 10 μl of the diluted solution was transferred to a Neubauer slide, and the spermatozoa per milliliter of sample were counted. To calculate the motility, the sperm were observed through an inverted microscope. The viability of the sperm was studied by Trypan Blue staining.

### 2.8 Sperm DNA fragmentation assessment

The sperm chromatin dispersion (SCD) test to evaluate the sperm DNA fragmentation levels was performed according to the protocol of Fernandez et al.^24^

### 2.9 Statistical analysis

To confirm a normal distribution of the data, Kolmogorov–Smirnov analysis was performed. Statistical analysis was conducted by one‐way ANOVA and Tukey’s post hoc test using SPSS software version 21, and a *p* < 0.05 was considered statistically significant.

## 3 RESULTS

### 3.1 FBS levels

**FIGURE 1:**
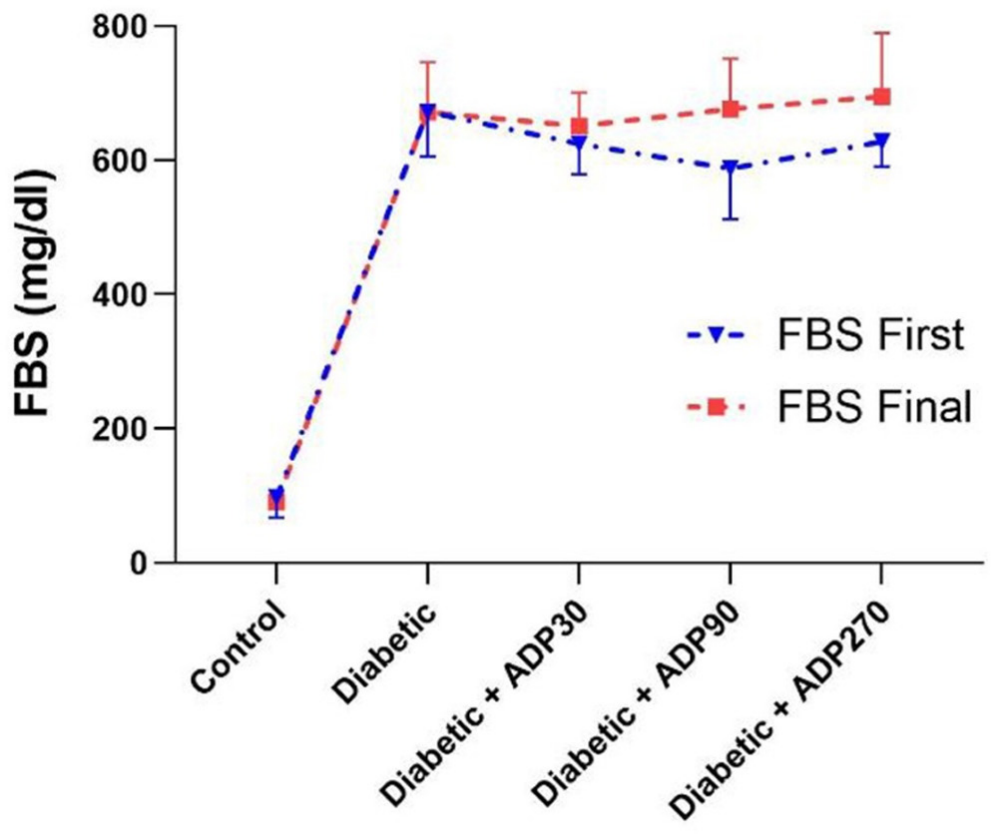
The levels of FBS in groups of control rats, diabetic rats and diabetic rats treated with various doses of ADP hydroalcoholic extract. Data were presented as mean ± SEM

Based on the results, no significant differences in FBS levels between any of the groups studied were observed before and after treatment with ADP hydroalcoholic extract (*p* > 0.05) (Figure 1).

### 3.2 Serum testosterone levels

**FIGURE 2:**
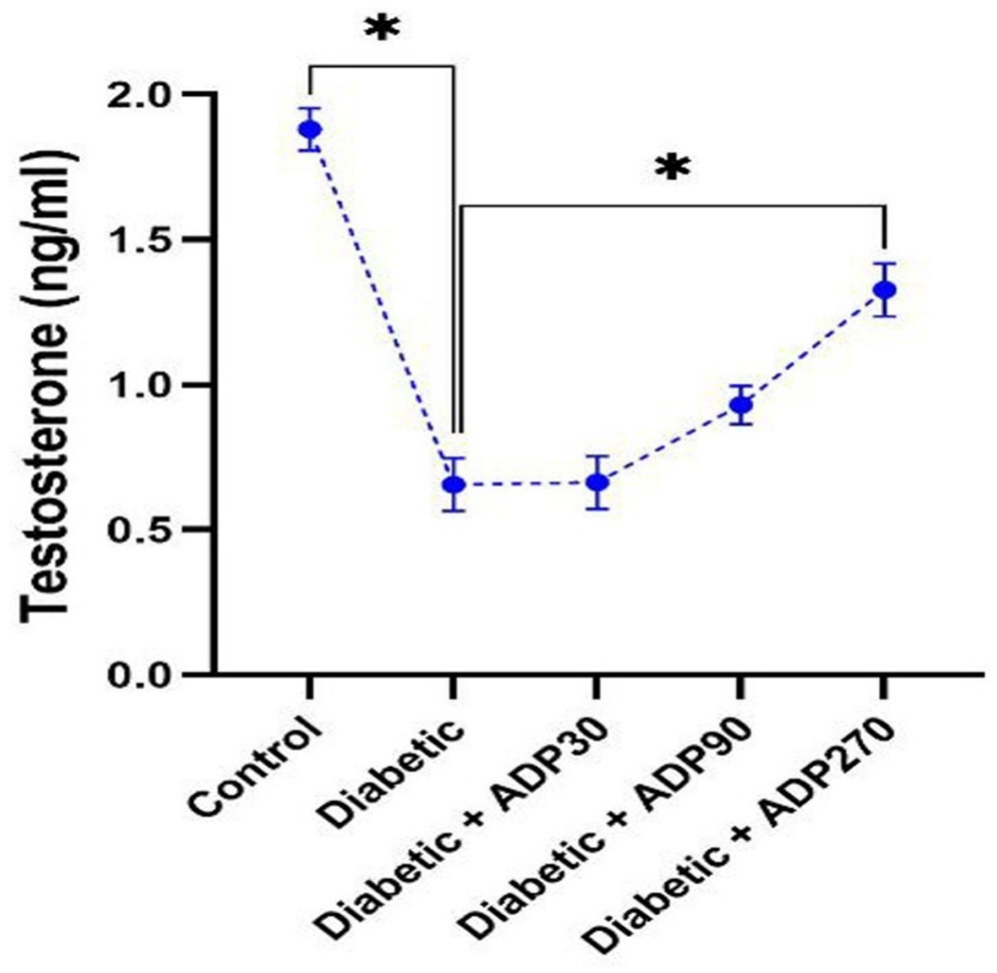
The levels of serum testosterone in all studied groups. A 270 mg/kg dose of ADP hydroalcoholic extract significantly increased serum testosterone in diabetic rats (*p* < 0.05) . Data were presented as mean ± SEM and * indicates *p* < 0.05

The induction of diabetes led to a considerable reduction in levels of serum testosterone in diabetic rats compared to the control rats. Treatment with ADP hydroalcoholic extract, especially at a dose of 270 mg/kg, can ameliorated the levels of serum testosterone (*p* < 0.05) (Figure 2).

### 3.3 Effects of various concentrations of ADP hydroalcoholic extract on sperm parameters

**FIGURE 3:**
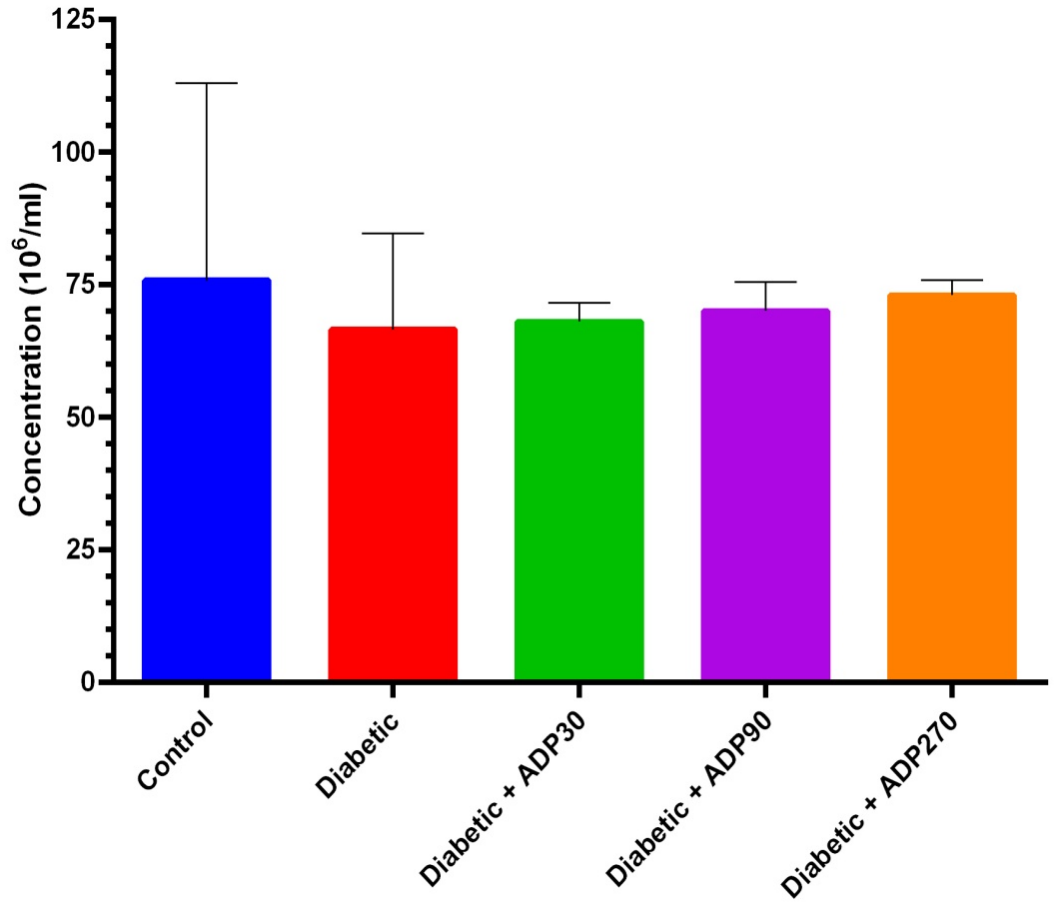
Sperm concentration in the groups of control rats, diabetic rats and diabetic rats treated with doses of 30, 90 and 270 mg/kg (Diabetic + ADP30, Diabetic + ADP90 and Diabetic + ADP270) of ADP hydroalcoholic extract. None of the doses of this extract had a significant effect on sperm concentration (*p* > 0.05)

In our investigation of the effect of the ADP hydroalcoholic extract on sperm parameters, we first evaluated the sperm parameters in groups of control and diabetic rats. The diabetic group showed significantly decreased viability, motility and sperm count during the study period compared with the control group. These alterations demonstrate that diabetes has adverse affects on the sperm parameters. In the present study, the doses of the ADP hydroalcoholic extract administered—30, 90 and 270 mg/kg—enhanced the sperm count per milliliter, but this increase was not significant (*p* > 0.05) (Figure 3).

**FIGURE 4:**
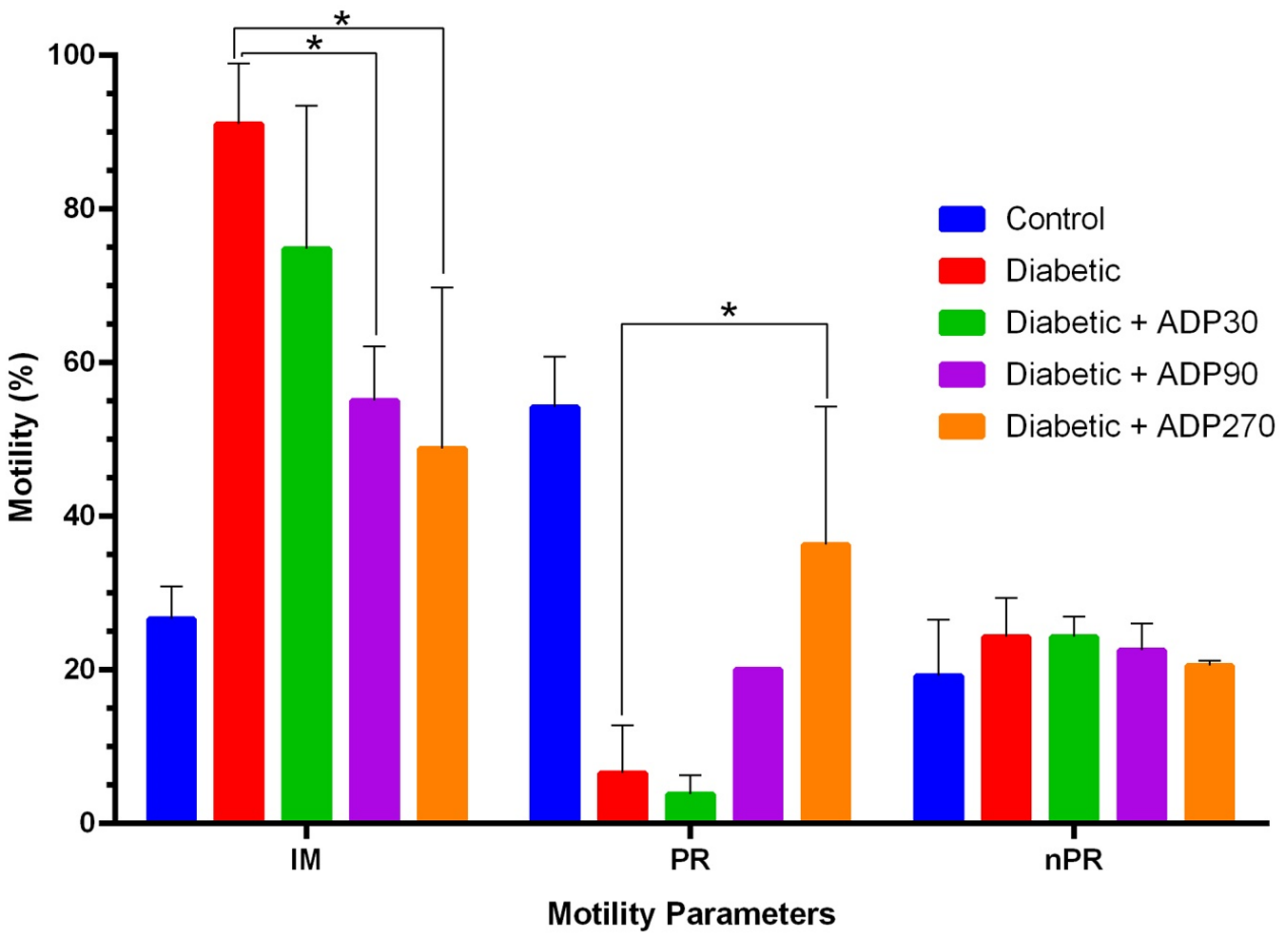
Sperm motility in the groups of control and experimental rats treated with doses of 30, 90 and 270 mg/kg of ADP hydroalcoholic extract. At doses of 90 and 270 mg/kg, ADP extract considerably reduced the number of immotile sperm (IM; *p* < 0.05), and at 270 mg/kg dose, significantly increased the forward sperm motility (PR; *p* < 0.05). At doses of 90 and 270 mg/kg, the extract decreased non‐progressive motility (nPR), but the decrease was not statistically significant (*p* > 0.05). Data were presented as mean ± SEM and * indicates *p* > 0.05

**FIGURE 5:**
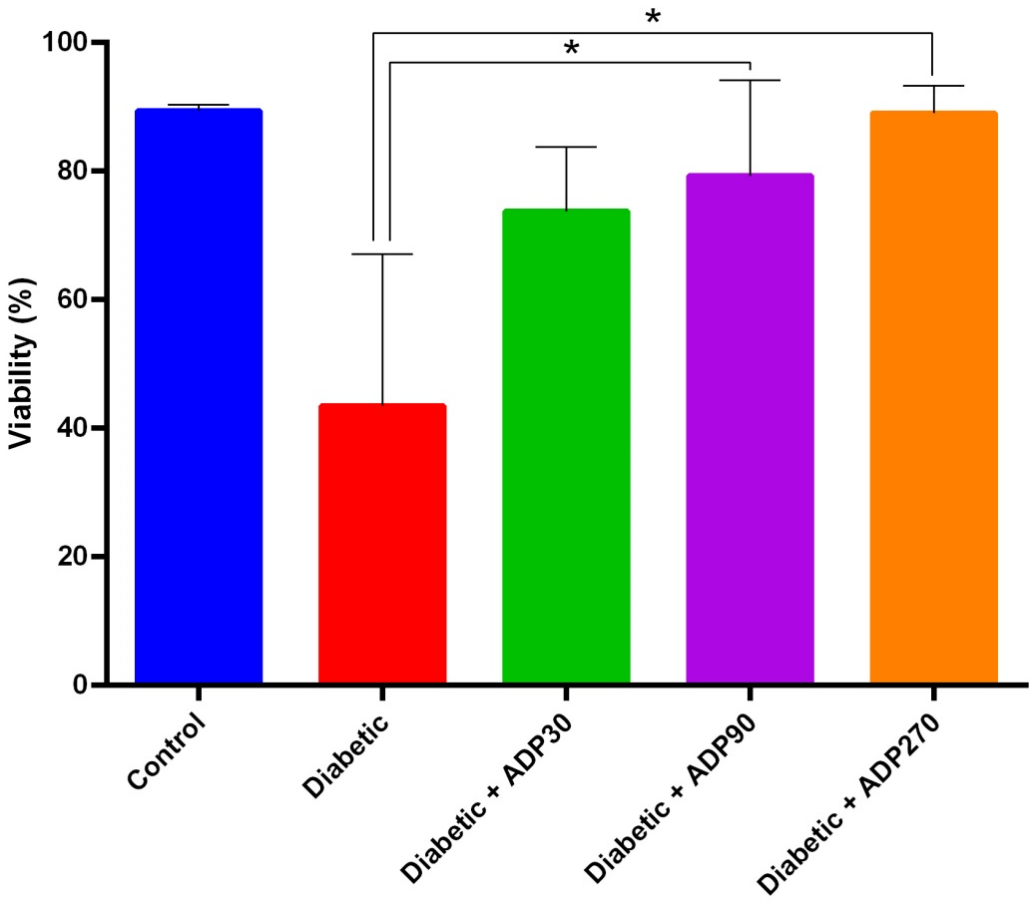
The effect of ADP extract on sperm viability. At doses of 90 and 270 mg/kg, ADP extract considerably increased the viability of sperm compared to the diabetic group (*p* < 0.05). Data were presented as mean ± SEM and * indicates *p* < 0.05

The 90 and 270 mg/kg doses of the ADP hydroalcoholic extract led to a significant improvement in sperm motility, with a significant decrease in immotile sperm (IM; *p* = 0.002 and *p* = 0.006, respectively) and, at the 270 mg/kg dose, a considerable enhancement of forward sperm motility (PR; *p* = 0.04) (Figure 4). Moreover, the 90 and 270 mg/kg doses of extract significantly increased sperm viability compared to the diabetic group (*p* = 0.04 and *p* = 0.03, respectively) (Figure 5).

### 3.4 Effect of different concentrations of ADP hydroalcoholic extract on sperm DNA fragmentation

**FIGURE 6:**
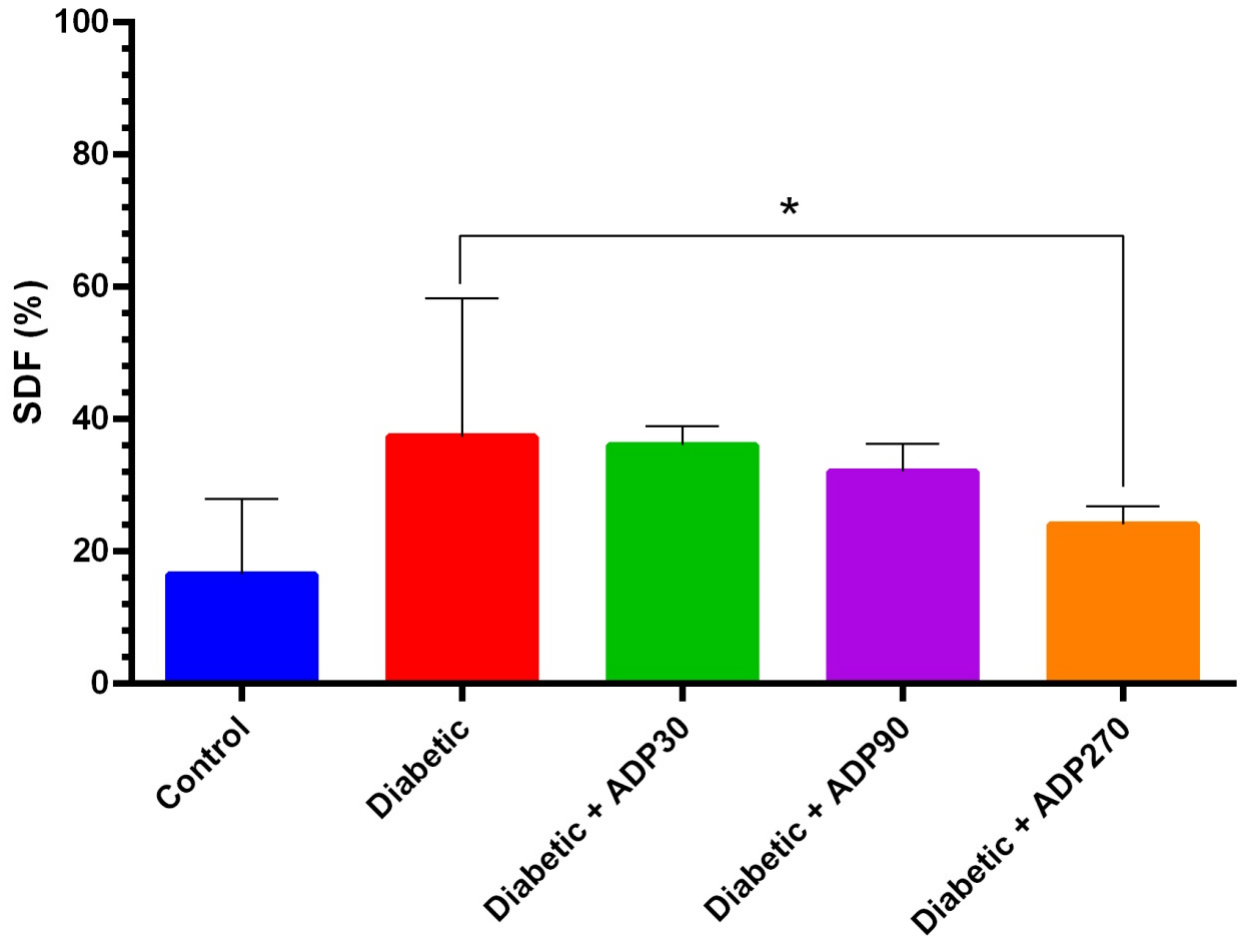
The effect of ADP extract on sperm DNA fragmentation. The groups treated with the various doses of ADP extract showed a reduction in levels of sperm DNA fragmentation, with the 270 mg/kg dose leading to the greatest decrease in sperm DNA fragmentation (*p* < 0.05). Data were presented as mean ± SEM and * indicates *p* < 0.05

Sperm DNA fragmentation (SDF) in all groups was assessed. The results of this test showed that induction of diabetes led to a significant enhancement in levels of sperm DNA fragmentation in the diabetic group compared to the control group. The various doses of ADP extract administered during 5 weeks showed significant effects on levels reduction of DNA fragmentation in the diabetic groups. Diabetic rats treated with a 270 mg/kg dose of ADP extract demonstrated the greatest decrease in sperm DNA fragmentation compared to the diabetic group (*p* = 0.04) (Figure 6).

## 4 DISCUSSION

Over‐production of ROS and diabetes‐induced oxidative stress can result in injury to the male reproductive system and the spermatogenesis process. Various factors can lead to male infertility, and over‐production of ROS is one of the commonest causes that its high levels is related to infertility in 30% to 80% of men.^25^, ^26^, ^27^, ^28^ In fact, high levels of ROS can have adverse effects on the morphology, motility, viability and concentration of sperm, as well as on sperm function and oocyte‐sperm fusion. It can also lead to lipid peroxidation in sperm plasma membrane, sperm DNA damage and apoptosis of testicular tissue cells.^3^, ^28^, ^29^, ^30^, ^31^ However, the apoptosis process in sperm cells can also be triggered by ROS‐independent pathways including FAS/FASL signaling and the mitochondrial pathway.^32^, ^33^ On the other hand, in the reproductive system under physiological conditions, regulated levels of ROS contribute to promoting vital intracellular signaling pathways such as capacitation, maturation, motility, acrosome reaction and fertilization.^34^, ^35^

There is evidence that the hydroalcoholic extract of ADP can reduce these complications via its antioxidant properties.^18^ Therefore, in the present study we evaluate, for the first time, the protective and antioxidant properties of ADP on sperm parameters and the integrity of sperm DNA in diabetic rats. Our findings demonstrated that the administration of ADP in diabetic rats significantly improved the sperm parameters as well as reducing the percentage of DNA fragmentation in sperm. We showed that the hydroalcoholic extract of ADP, especially at a dose of 270 mg/kg, considerably enhanced serum testosterone levels (*p* < 0.05). The 90 and 270 mg/kg doses of the ADP hydroalcoholic extract also resulted in a significantly decrease in immotile sperm (*p* < 0.05), while a 270 mg/kg dose considerably enhanced forward sperm motility (*p* < 0.05). Moreover, sperm viability significantly increased compared to the diabetic group (*p* < 0.05) with doses of 90 and 270 mg/kg of the extract. No significant effect on sperm count was seen at any of the treatment doses of 30, 90 and 270 mg/kg of extract. The various doses of extract administered demonstrated significant effects on levels of sperm DNA fragmentation in diabetic rats, and a 270 mg/kg dose of ADP extract led to the greatest rate of decrease in DNA fragmentation compared to the diabetic group (*p* < 0.05). ADP has been shown to have potent antioxidant activity that can inhibit ROS production. In our previous study, we showed that STZ‐induced diabetes led to biochemical and structural changes in the testicular tissue and ADP extract, due to its antioxidant effects, can reverse these testis alterations and restore the levels of testosterone and the activities of antioxidant enzymes such as CAT, SOD and GPX towards normal levels.^18^

The findings of our LC–MS analysis show that there are eleven chemical components in the hydroalcoholic extract of ADP. The most prevalent of these compounds were methyl esters of 9‐octadecenoic acid (Z) and dodecanoic acid, and previous studies have confirmed the antioxidant properties of these compounds.^18^ STZ‐induced diabetes can lead to enhancement of MDA activity and lipid peroxidation, and it seems that antioxidant enzymes such as SOD, CAT and GPx were suppressed in testis tissue. These alterations might result in apoptosis and the loss of Leydig and Sertoli cells. Dysregulation of apoptosis also can disrupt the process of spermatogenesis and affect sperm parameters. The findings of the present study are in agreement with the results of some other studies. For example, Jalili et al. showed that *Falcaria vulgaris* extract has antioxidant effects in diabetic rats and can improve their sperm parameters.^36^ A study by Ahangarpour et al. found that extract of applied burdock root has anti‐infertility effects via enhancement of sperm count, levels of testosterone and gonadotropin in nondiabetic mice.^37^ Another study demonstrated that hydroalcoholic extract of *Alpinia officinarum* can ameliorate the sperm parameters in STZ‐ induced diabetic rats, probably via its antioxidant components.^38^

Roshankhah et al. demonstrated the protective effects of the ADP hydroalcoholic extract against mercuric chloride‐induced liver damage. They reported that treatment with ADP, due to its potent antioxidant properties, can result in decreased ROS, the activation of antioxidant factors and detoxification enzymes.^19^

Overall, based on our findings and previous studies, it seems that the antioxidant compounds which exist in an ADP hydroalcoholic extract can protect testis tissue against injury caused by free radicals and improve sperm parameters and the amount of sperm DNA fragmentation. Therefore, this extract, with its antioxidant properties, can be considered as an alternative useful compound for treating oxidative injury induced by diabetes.

In summary, the hydroalcoholic extract of ADP through its antioxidant properties can improves the injury caused by diabetes in testicular tissue. The most important compounds identified in this extract were the methyl esters of 9‐octadecenoic acid (Z) and dodecanoic acid, which have potent antioxidant effects. Therefore, this extract can improve sperm parameters and protect the sperm DNA integrity in diabetic animals. Further experimental studies are required to determine the exact mechanism of action of ADP hydroalcoholic extract in the male reproductive system.

## Ethical statement

All experimental protocols were performed based on the Ethics Committee of Kermanshah University of Medical Science (approval no: IR.KUMS.REC. 1397.793).

## CONFLICT OF INTEREST

The authors report no conflicts of interest.

## AUTHOR CONTRIBUTIONS

Conception and design: Mitra Bakhtiari and Morteza Hosseinipour; Development of methodology: Morteza Hosseinipour, Nader Goodarzi and Mitra Bakhtiari; Acquisition of data: Morteza Hosseinipour, Javid Kermani, and Mitra Bakhtiari; Analysis and interpretation of data: Mitra Bakhtiari, and Nader Goodarzi; Writing and revision of the manuscript: Mitra Bakhtiari and Rezvan Asgari. All authors read and approved the final manuscript.
